# Does retrieval practice protect memories against the amnesic effects of stress? An evaluation of the role of stimulus difficulty

**DOI:** 10.3389/fcogn.2026.1838176

**Published:** 2026-06-17

**Authors:** Cadu Klier, Luciano Grüdtner Buratto

**Affiliations:** Department of Basic Psychological Processes, Institute of Psychology, University of Brasília (UnB), Brasília, Brazil

**Keywords:** cortisol, item difficulty, memory, retrieval practice, stress

## Abstract

**Introduction:**

Previous research suggests that the protective effect of retrieval practice against acute stress may be modulated by item difficulty, with difficult items showing distinct vulnerability patterns compared to easy items. The present study aimed to replicate and extend these findings by strictly controlling for initial memory strength—a potential confound in prior work.

**Methods:**

Using a 2 (Stress: Control vs. Stress) × 3 (Difficulty: E4, D4, D8) mixed factorial design, 56 male participants encoded 48 Swahili-Portuguese word pairs via retrieval practice. Items were manipulated by memorability and practice dosage to equalize initial strength between Easy (E4) and Difficult-Extended (D8) conditions. Forty-eight hours after encoding, participants underwent the modified Socially-Evaluated Cold Pressor Test (SECPT) or a control procedure before a final cued-recall test.

**Results:**

Although the stress manipulation successfully elevated salivary cortisol and autonomic measures, the previously observed Stress × Difficulty interaction was not replicated. Results indicated a main effect of Difficulty, but stress did not impair retrieval performance across conditions. Notably, difficult items subjected to extended practice (D8) achieved recall rates comparable to easy items (E4), and both were unaffected by stress.

**Discussion:**

These findings suggest that when memory traces are strengthened via repeated retrieval practice, they may become resistant to the deleterious effects of acute stress, regardless of intrinsic item difficulty.

## Introduction

Finding effective study strategies to protect memory against deleterious effects would have important implications in applied educational contexts, as it would suggest the use of such strategy to reduce the “blank-outs” that occur in situations like oral presentations, interviews or during important exams. Some of the most popular study strategies consist of attentively reading and rereading the material one seeks to learn, underlining important passages of the material, and even elaborating conceptual maps, with consultation, relating the concepts studied (Ekuni et al., 2022; Karpicke, 2009). Nevertheless, empirical studies have repeatedly shown that a technique as simple as closing the book and trying to remember the maximum amount of information present in the material to be learned ends up having a more durable and more effective effect for future inferences than attentive, repeated reading, with underlined passages and with consultation-based conceptual map elaboration (Karpicke and Blunt, 2011; Karpicke and Roediger, 2008; Rowland, 2014). This phenomenon, which involves retrieval effort, is called the testing effect or the Retrieval Practice effect, and it has important implications for clinical (Lima et al., 2020a) and educational practice (Dunlosky et al., 2013; Rowley and McCrudden, 2020). The Retrieval Practice effect is quite robust, occuring even without feedback (Roediger and Karpicke, 2006) and having been reported even under conditions where the encoding or retrieval of information is degraded. For example, the effect is found when attention is divided during the Retrieval Practice phase (when the learner practices the retrieval of the materials of interest) (Buchin and Mulligan, 2017). The effect is also found when the final recall test occurs in a situation of acute psychosocial stress (Smith et al., 2016; Szollosi et al., 2017).

To understand why retrieval practice might offer such robust protection, it is necessary to look at the mechanisms underlying successful learning, particularly the role of effort. Various factors modulate the Retrieval Practice effect, such as differences in fluid intelligence among participants (Lima and Buratto, 2025; Minear et al., 2018) and differences in encoding conditions that result in easier or more difficult retrieval attempts (e.g., when the intervals between successive retrieval attempts are shorter or longer; Pyc and Rawson, 2009). In the context of Retrieval Practice, effort can be operationalized in terms of the memorability of a Swahili-Portuguese pair (Nelson and Dunlosky, 1994), based on norms that indicate the probability of recalling the target after studying the cue-target. Norms for Swahili-Portuguese pairs were validated in Brazil (Lima and Buratto, 2021) and similar norms have been used in studies in different laboratories to evaluate the Retrieval Effort Hypothesis (Lima et al., 2020b; Minear et al., 2018; Vaughn et al., 2013). Conditions that make recall more difficult are of particular interest because they allow testing an influential explanation for the Retrieval Practice effect. According to the Retrieval Effort Hypothesis (REH), successful retrievals that are more difficult to perform result in more durable memory traces than easier-to-perform successful retrievals (Bjork and Bjork, 1992; Pyc and Rawson, 2009). In an experimental setting, the concept of effort can be operationalized by choosing materials with varied levels of intrinsic difficulty of memorization (“easy” vs. “difficult”; see below). Why would more difficult retrievals be more beneficial than easy retrievals for long-term retention? According to the REH, elaborated in Bjork and Bjork's (1992) new theory of disuse, items are represented in memory by two types of strength: the first is a storage strength, which reflects the intensity of the relatively permanent trace of a representation in memory, and the second is a retrieval strength, which indicates the momentary accessibility of that representation in memory at a given moment. Repeated study of content (restudy) would increase both storage strength and retrieval strength (traces become stronger in memory and more accessible). Repeated Retrieval Practice, however, would further increase the storage strength and retrieval strength of the items in memory. This difference in the increments of strength on the memory trace and its accessibility would help explain the Retrieval Practice effect. More importantly, items with low retrieval strength (i.e., those that are harder to recall; with low memorability) benefit more from a correct retrieval than items with high retrieval strength (i.e., those that are easier to recall; with high memorability). In summary, the Retrieval Effort Hypothesis predicts a greater benefit of Retrieval Practice for more difficult items because these items would have more to gain in terms of memory trace strength.

Beyond intrinsic item memorability, environmental and physiological factors can also significantly modulate retrieval success. One manipulation that can increase the difficulty of item retrieval is the induction of acute psychosocial stress a few minutes before a final recall test (Shields et al., 2017). Stress induction protocols, such as the Socially-Evaluated Cold Pressor Test (SECPT; Schwabe et al., 2008) can reliably activate the hypothalamus-pituitary-adrenal axis, resulting in an increase of the hormone cortisol in the bloodstream (Schwabe and Schachinger, 2018). Circulating cortisol binds to receptors in the hippocampus and the amygdala, brain areas crucial for the encoding and retrieval of episodic memories, interfering with their functions (Kim et al., 2006; Yassa and Stark, 2011). Approximately 30 min after the onset of the stress induction protocol, cortisol reaches peak levels in the bloodstream, and consequently, the recall of previous events is impaired to a greater degree than when the recall test occurs immediately after the end of the stress induction protocol (Schwabe and Wolf, 2014; Shields et al., 2017).

Given the intersection between these two factors, researchers have investigated whether Retrieval Practice can buffer memory performance against cortisol-induced retrieval deficits. In an influential study, Smith et al. (2016) presented evidence that Retrieval Practice, if used as a learning method, could eliminate the detrimental effect of stress on free recall compared to the restudy control condition. The results of Smith et al. (2016), however, have been difficult to replicate. Szollosi et al. (2017) attempted to replicate and extend Smith et al.'s findings by manipulating the study strategy within-subjects, instead of between-subjects as in Smith et al. (2016); by using a longer retention interval of 7 days instead of 24 h; and by assessing learning and retention via cued recall with feedback (Swahili-Hungarian pairs) in place of testing word lists and images via free recall without feedback. Although Szollosi et al. (2017) obtained a significant Retrieval Practice effect, they failed to replicate the protective effect of Retrieval Practice against stress. The stress manipulation impaired the recall of pairs studied via Retrieval Practice as well as the recall of pairs studied via restudy. In a subsequent study with Swahili-English pairs and cued recall, Smith et al., 2018a also failed to replicate their original finding with free recall. Despite a large effect in cued recall after Retrieval Practice, performance on the final cued-recall test 7 days after initial encoding was nevertheless impaired by the psychosocial stress induction. The inconsistencies may be related to the different protocols used, which may generate direct or indirect benefits of Retrieval Practice on the final test. In Smith et al. (2016), participants performed Retrieval Practice via free recall and without feedback (the classic Retrieval Practice effect), and Retrieval Practice protected performance against the amnesic effects of stress. In Szollosi et al. (2017) and (Smith et al., 2018a), on the other hand, participants completed free-recall tests with feedback (Arnold and McDermott, 2012), and Retrieval Practice did not completely protect performance against the amnesic effects of stress. Therefore, the protective role of Retrieval Practice against the amnesic effects of acute psychosocial stress is still uncertain.

A potential explanation for these mixed findings lies in the intrinsic difficulty of the materials used across different studies. In a recent study, we showed that the difficulty of the learned item can be a moderator of the effect of stress on memory (Klier and Buratto, 2023), which could help explain the inconsistencies observed in previous studies. Swahili-Portuguese word pairs were divided into “easy” and “difficult” based on their memorability norms (Lima and Buratto, 2021). Both types of pairs were learned and practiced either via Retrieval Practice (cued recall with feedback) or via restudy. After 7 days, participants returned for the final cued-recall test. Before the test, participants underwent either the stress induction protocol (modified SECPT; see description in the Method below) or the control protocol. The final test occurred 25 min after the onset of the stress induction protocol to maximize cortisol concentration in the blood at the time of the test. The most relevant result refers to the proportion of correct responses (i.e., correctly produced targets) as a function of item difficulty (Easy vs. Difficult) and Group (Control vs. Stress). The result shows a Difficulty × Group interaction, indicating that the effect of stress on cued recall was observed for easy items (lower recall in the Stress condition), but not for difficult items (higher recall in the Stress condition).

An important point about the result is that it refers to items correctly recalled at least once during the Retrieval Practice phase (conditional analysis). This restriction is important because the Retrieval Practice effect is strongly modulated by the success of retrieval during practice (Karpicke and Roediger, 2007). This finding may help explain the discrepancies noted in the literature because previous studies used a mixture of high and low memorability items. It is possible that easy items, which are more sensitive to the effects of stress, had their effect nullified by difficult items, which are less sensitive to the effects of stress. By more rigorously separating and controlling the difficulty of the studied items, it would be possible to better understand the role of Retrieval Practice in the context of acute psychosocial stress.

Despite these promising results, the relationship between retrieval effort and stress might be confounded by initial learning levels. Klier and Buratto (2023) indicated an interesting role of item difficulty as a modulator of the effect of stress on memory. However, the study presented some limitations. Here we propose a modification to address one of these limitations. During the Retrieval Practice phase, easy items tend to be recalled more often than difficult items. This is a natural consequence of the stimulus selection (i.e., easy items are easy because they present memorability norms indicating faster learning). Throughout the test-study cycles, the difference in the proportion of items recalled remains consistently greater for easy items than for difficult items. Since the number of correct retrievals during the practice phase is a powerful modulator of the Retrieval Practice effect (Karpicke and Roediger, 2007; Rowland, 2014), easy items end up benefiting more than difficult items both during the practice phase and in the final test (i.e., main effect of difficulty: greater recall of easy items than of difficult items). This benefit for easy items is not necessarily due to retrieval effort—the retrieval of easy items requires less effort, operationalized by the higher percentage of correct responses after the first presentation and the shorter response time.

To directly address this confound, the present study was designed to isolate the effect of effort by controlling initial memory strength. We designed the experiment so that the proportion of correct responses during the Retrieval Practice phase between easy and difficult items would be similar. With this proportion equalized, the impact of item difficulty on the final recall test 2 days later could then be assessed. In this way, the Difficulty factor has now three levels instead of two: easy items with 4 practice blocks, difficult items with 4 practice blocks, and difficult items with 8 practice blocks. The study was a replication of the previous one because we expected to obtain a result similar to that obtained for easy and difficult items with 4 practice blocks. The study was also an extension because it allowed us to evaluate whether Retrieval Practice, for higher performance levels for difficult items (D8) and comparable to those of easy items, maintained its protective factor against the amnesic effects of stress. We did not include a restudy condition because our hypothesis was restricted to the relationship between retrieval practice and item difficulty.

## Method

### Participants

Fifty-six young, healthy men were recruited from the University of Brasília student body to participate in the study. Participants were randomly allocated to one of two Groups: 26 to the Control condition and 30 to the Stress condition. Initially, 61 participants were recruited, but four were excluded due to familiarity with Swahili, and one was excluded for not being a native Portuguese speaker. Participants were included only if they had abstained from coffee and energy drinks for at least 2 h before the session, from smoking and eating for at least 3 h, from alcoholic beverages for at least 12 h, and from illegal substances for at least 24 h before the session.

Women did not participate to avoid the confounding effect of the menstrual cycle period on blood cortisol concentrations. Volunteers were native Portuguese speakers and had no prior knowledge of the Swahili language. Volunteers with a history of neurological or psychiatric disorders and who were taking psychotropic medication did not participate in the study. Before starting the experiment, participants read and signed an Informed Consent Form. This research and all protocols used within it were submitted to and approved by the Ethics Committee for Research in Human and Social Sciences in Brazil.

Previous studies in the area investigating similar variables have used samples of 60 participants (30 in each Group) (Smith et al., 2016, 2018a; Szollosi et al., 2017). We calculated the sample size for the present study using G^*^Power (v. 3.1.9.6; Faul et al., 2007). The analysis was based on a 2 × 3 mixed ANOVA, with Stress (Control vs. Stress) manipulated between-subjects and Item Difficulty (E4, D4, D8) manipulated within-subjects. The effect size of the interaction of interest (Stress vs. Difficulty) in Klier and Buratto's (2023) study was ${\;\eta }_{p}^{2}$ = 0.15, which is a high value. Considering Type I (alpha) and Type II (beta) error probabilities of 0.05, and a conservative correlation value of 0.50 between the levels of the within-subjects variable (Difficulty), we obtained an indication of a total sample of 18 participants to achieve 95% power to detect the effect of interest. This is a very low value, resulting from the high effect size found previously. To better assess the replicability of the finding, we recruited a larger sample (*N* = 60), following the more common practice in this literature.

### Experimental design

The study employed a 2 (Stress: Control vs. Stress) × 3 (Difficulty: E4, D4, D8) mixed factorial design. The first independent variable, Stress, was manipulated between-subjects and refers to the protocol used to induce acute psychosocial stress (modified SECPT) or the control task. The second independent variable, Difficulty, was manipulated within-subjects and refers to the assignment of word pairs into three conditions based on item memorability and retrieval practice dosage (Easy items with standard practice [E4], Difficult items with standard practice [D4], and Difficult items with extended practice [D8]). Regarding the dependent variables, memory performance was measured by the proportion of correctly recalled targets in the final cued-recall test. The physiological stress response was assessed via heart rate (HR), systolic and diastolic blood pressure (SBP/DBP), and salivary cortisol concentrations. Finally, subjective stress was measured using the stress subscale of the Depression, Anxiety, and Stress Scale (DASS-21).

### Materials and tasks

#### Stimuli

To operationalize retrieval effort, the stimulus set consisted of 48 Swahili-Portuguese word pairs selected from the normative database by Lima and Buratto (2021). In this type of paradigm, the goal is to simulate foreign vocabulary learning. For instance, the first word, wingu, is in Swahili, a language spoken in East African countries and with phonological and orthographic similarities to Portuguese, but with low semantic similarity and low familiarity among Portuguese speakers in Brazil. The participants were asked to memorize that wingu means nuvem (cloud).

To accommodate the experimental conditions, the stimuli were organized into three lists based on their average memorability scores. The High-Memorability List (assigned to condition E4) comprised 16 pairs with high recall probability (*M* = 0.55, *SD* = 0.14). The Low-Memorability Lists (assigned to conditions D4 and D8) comprised two balanced sets of 16 pairs each, with low recall probability (*M* = 0.28, *SD* = 0.07 for List D4; and *M* = 0.29, *SD* = 0.07 for List D8). Analysis of variance confirmed the distinction between high and low memorability lists (*p* < 0.001). Crucially, the three lists were balanced across psycholinguistic dimensions. No significant differences were found regarding target familiarity, concreteness, valence, arousal, wordlikeness, word length (both languages), or lexical frequency (all *ps* > 0.52). Additionally, the semantic content was balanced, with an equal distribution of items classified as foods, animals, concepts, and objects across all lists (*p* = 0.90).

#### Stress induction protocol (modified SECPT)

Stress was induced using a modified version of the Socially-Evaluated Cold Pressor Test (SECPT), a simple, effective, and widely used procedure to activate the hypothalamus-pituitary-adrenal axis, resulting in increased blood cortisol levels detectable via saliva testing (Schwabe et al., 2008; Schwabe and Schachinger, 2018).

In the modified version of the SECPT, participants were instructed to submerge one hand in a container with ice water (0–2°C) for 1 min to 2 min. The apparatus, called the cold pressor, allows for fine control of the water temperature. In addition to the physiological induction of stress, produced by the cold water, the SECPT protocol has a psychosocial component: Participants were instructed to look directly at a camera present in the room and were informed that a video would be recorded and that their facial expressions would be evaluated later by a Group of researchers. After the hand submersion period, participants were instructed to watch a 20 min video from the series Amazônia (this is a relatively neutral stimulus, used in previous stress studies; e.g., Smith et al., 2018a). During the episode (3 min after the start), participants were instructed to perform a mental subtraction task out loud in front of the experimenter. This task is not part of the original SECPT protocol, but it is part of another widely used stress induction protocol called the Trier Social Stress Test (TSST; Allen et al., 2017; Kirschbaum et al., 1993). The mental subtraction task adds another element of social evaluation and unpredictability to the original SECPT protocol. This modified version of the SECPT protocol strongly activates the hypothalamus-pituitary-adrenal axis (Boyle et al., 2016).

In the control version of the modified SECPT protocol, participants performed similar tasks with the exception that the water where they submerged one hand was warm (23–25 °C) and there were no elements of social evaluation (i.e., no video recording and no mental subtraction). The experimenter remained in the room to ensure compliance with instructions. Although the stress induction protocol seems uncomfortable, none of the participants dropped out of the previous experiment (Klier and Buratto, 2023). The only exclusions were due to failures in salivary cortisol collection and non-compliance with task instructions. The application of the modified SECPT protocol (stress condition) generated a large increase in cortisol concentration compared to the control condition (Klier and Buratto, 2023).

#### Physiological measures

Blood pressure (systolic and diastolic) and heart rate were measured with an automatic blood pressure monitor (HEM-7130; Omron Healthcare Brazil, São Paulo). Salivary cortisol samples were collected with a chewable synthetic swab (Sarstedt Cortisol Salivette^®^ code blue; Nümbrecht, Germany), an indicator of Sympathetic Nervous System activation.

#### Depression, anxiety, and stress self-report questionnaire (DASS-21)

Subjective stress was measured with the DASS-21, a 21-question, multiple-choice, self-report inventory used for measuring depression, anxiety, and stress, used in research and validated for use in Brazil (Vignola and Tucci, 2014). The results presented refer to the 7 items of the questionnaire that specifically deal with stress (stress subscale).

### Procedure

#### General setup

**Figure 1:**
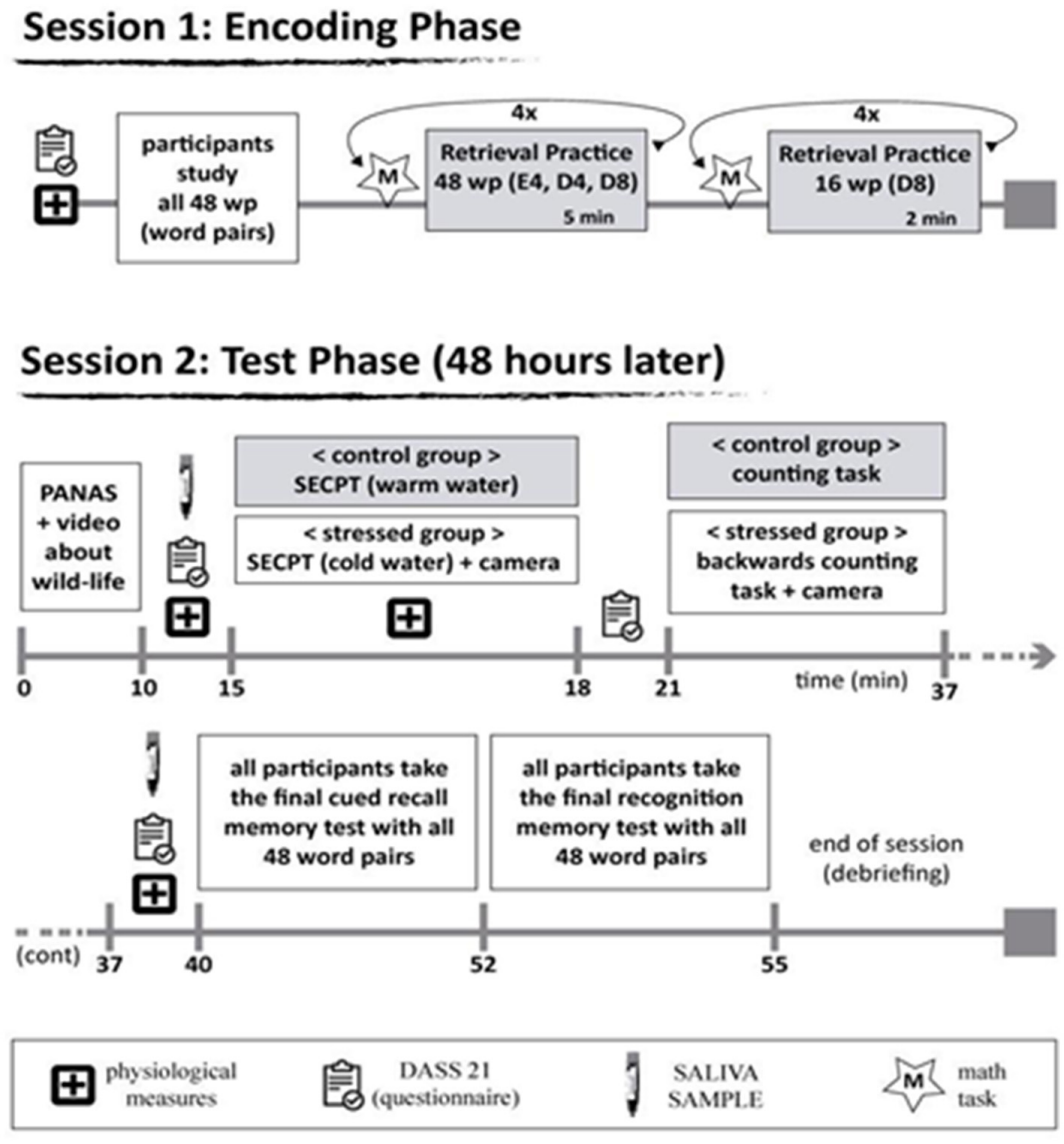
Experimental sessions. E4: Easy items presented in 4 test-study cycles; D4: Difficult items in 4 test-study cycles; D8: Difficult items in 8 test-study cycles. DASS-21: Depression, anxiety and stress scale. Cortisol collected along with other physiological measures (e.g., heart rate, systolic pressure) before and after the stress protocol. The math task consisted of simple arithmetic operations.

The participants attended two sessions (Figure 1). Session 1 (Encoding Phase) comprised the encoding task and the test-study cycles (Retrieval Practice with feedback). Session 2 (Test Phase) occurred 2 days later and comprised the experimental intervention (stress induction protocol or its control) and the final cued-recall test. Sessions were scheduled to always occur between noon and 5 p.m. due to the circadian variations of cortisol. The sessions were conducted in the same room, equipped with air conditioning, the cold pressor apparatus, and a computer. To ensure precise timing and stimulus presentation, data collection was implemented in PsychoPy (Peirce et al., 2019).

In the Encoding Phase, participants signed an Informed Consent Form, had their physiological measures collected (HR0, SBP0, DBP0) and filled out the DASS-21 questionnaire (DASS0). Following this, participants were exposed to 48 Swahili-Portuguese word pairs, presented in random order on the computer screen, with each pair presented for 6 s. Participants were instructed to read the presented pairs carefully. After this first presentation of the pairs, participants completed a series of test-study cycles (Retrieval Practice with feedback) based on the assigned difficulty condition. Items from the Easy (E4) and Difficult (D4) lists were presented in four test-study cycles. In contrast, items from the D8 list were presented in eight test-study cycles: the items presentation was interleaved with the other lists during the first four cycles and then continued for four additional cycles. In each trial, participants were required to type the Portuguese translation as soon as they saw the Swahili word (i.e., Retrieval Practice). Participants had 4.5 s to recall the target (i.e., Portuguese translation of the Swahili word presented on the screen) and type it. After this interval, the correct target was presented for 1.5 s, as feedback. One test-study cycle comprised 48 trials (16 pairs x 3 lists in random order). After completing a cycle, participants completed a Math Task with only a distractor function (simple arithmetic operations). After completing four cycles, participants completed four more cycles with the items from List D8. At the end of the session, participants were scheduled for the next session (Test Phase), which occurred 48 h later.

In the Test Phase, participants again had their physiological measures collected (HR1, SBP1, DBP1, SC1) and filled out the DASS-21 questionnaire (DASS1). Next, participants completed either the stress condition (modified SECPT protocol) or the control protocol. During the application of the protocol (control or stress), participants had their physiological measures collected (HR2, SBP2, DBP2) and, after removing their hand from the cold pressor, filled out the DASS-21 questionnaire once more (DASS2). The interval between the onset of the stress induction protocol and the start of the cued-recall test was 25 min. This interval is motivated by research showing that an interval of about 25 min is necessary for blood cortisol concentration to reach its peak in humans (Joels et al., 2011; Schwabe and Wolf, 2014). After the interval, participants filled out the DASS-21 questionnaire for the last time and had their physiological measures collected again (HR3, SBP3, DBP3, SC2). Following this, the final cued-recall test began. Cues (i.e., Swahili words) were presented on the computer screen, one at a time, for 15 s each. During this time, participants should type the target (i.e., Portuguese translation) as soon as they could recall it. Data collection was implemented in PsychoPy (Peirce et al., 2019).

#### Data analysis

A confidence level α of 0.05 was used for all statistical tests. Measures of effect size were reported as Cohen's *d* (in *t*-tests) or ${\;\eta }_{p}^{2}$ (in ANOVAs).

##### Encoding phase

To evaluate learning trajectories during the first session, paired-samples *t*-tests assessed recall performance between the first and final practice cycles within each condition. Similarly, we compared final-cycle performance across the different difficulty conditions (E4, D4, D8).

##### Stress manipulation check

To verify the efficacy of the modified SECPT, we first compared baseline physiological and subjective measures—including water temperature—between groups using independent *t*-tests. We then analyzed autonomic responses (heart rate, systolic and diastolic blood pressure) and subjective stress (DASS-21) via 2 (Group: Control vs. Stress) × 3 (Time: Pre-SECPT, During-SECPT, Post-SECPT) mixed ANOVAs. A separate 2 × 2 mixed ANOVA assessed salivary cortisol concentrations. Finally, participants were classified as cortisol responders (post-stress increase ≥ 2.5 nmol/L) or non-responders. Their distributions across groups were evaluated using a chi-square (χ^2^) test.

##### Final memory performance

Primary outcomes from the cued-recall test were evaluated using 3 (Difficulty: E4, D4, D8) × 2 (Group: Control vs. Stress) mixed-factorial ANOVAs. To isolate the effects of stress on established memory traces, we performed this analysis twice. The first evaluated the traditional proportion of total words recalled. The second used a conditionalized proportion, restricting the calculation solely to items correctly retrieved at least once during the Encoding Phase, thereby controlling for intrinsic memorability.

## Results

Data represent 56 participants who met the study's inclusion and exclusion criteria. The analyses were divided into analyses of the Encoding Phase, when participants learned the Swahili words, and Test Phase analyses, 48 h later, when their long-term memory was tested under conditions with or without induced stress.

### Encoding phase (session 1)

#### Target recall during practice blocks

In the Encoding Phase, the main performance measure was the proportion of Swahili words that participants managed to retain from one test block to another. There were three conditions: E4 (easy items with four repetition blocks), D4 (difficult items with four repetition blocks), and D8 (difficult items with eight repetition blocks). The objective of the D8 condition was to try to reach a performance level similar to that of E4 and, in this way, compare the effect of stress on memory under more similar recallability conditions (in D4 there was much forgetting, which made comparisons difficult).

**Figure 2:**
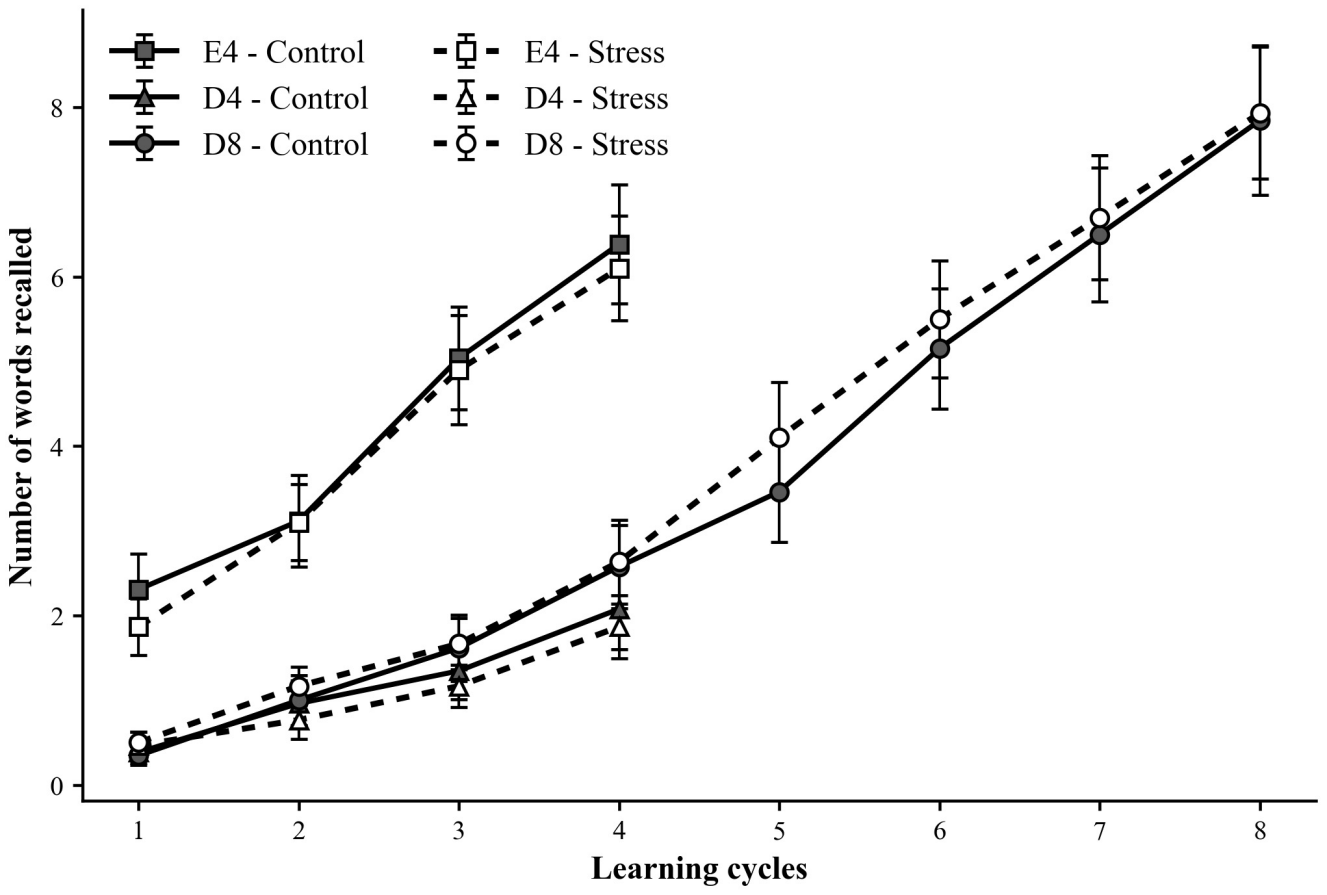
Number of words recalled as a function of learning cycle and tress condition. The learning of E4 and D8 items was greater than that of D4 items, and D8 items were recalled more in the final cycle than E4 items. Bars represent the standard error of the mean.

Figure 2 illustrates the learning curves across blocks for the three conditions. There were no differences between the control and Stress Groups. In this Encoding Phase, participants from both Groups were treated identically, so no difference was expected. For each item difficulty condition (E4, D4, D8), there was a progressive increase in the number of words recalled across the learning cycles. The differences between the last and the first cycle were significant in all conditions (*ts* > 6.32, *ps* < 0.001, *ds* > 0.13). As expected, the recall rate in the final cycle was higher in the E4 and D8 conditions than in the D4 condition, *ts* > 13.13, *ps* < 0.001, *ds* > 1.75.

The new condition of difficult items added to the study (D8) was successful in generating greater learning than the other difficult-item condition practiced fewer times (D4), *t*(55) = 13.13, *p* < 0.001, *d* = 1.76. In other words, difficult items practiced several times resulted in superior learning to difficult items practiced fewer times. The overall performance of the participants was low. Out of 48 word pairs trained, the final average in the last cycle in E4 and D8 was 8 pairs.

### Test phase (session 2)

#### Baseline physiological measures

First, we evaluated heart rate, systolic pressure, diastolic pressure, and the stress subscale questionnaire. There were no significant baseline differences between the control and Stress Groups (*ts* < 1, *ps* > 0.48), except in the diastolic pressure measure, which was higher for the Stress Group than for the Control Group, *t*(54) = −2.54, *p* = 0.04, *d* = −0.57. We conclude that the Groups were relatively homogeneous in these variables before the introduction of the manipulations.

#### Physiological measures during stress induction and post-stress

Next, we evaluated how the measures varied when the stress induction protocol was put into action. The second measures (HR2, SBP2, DBP2, DASS2) represent the moment when the participant's hand was immersed in the ice water. Table 1 summarizes these results.

**Table 1:** Physiological and self-report measures during the test phase (session 2).

Measures	Control (*N* = 26)			Stress (*N* = 30)		
	Pre-SECPT	During-SECPT	Post-SECPT	Pre-SECPT	During-SECPT	Post-SECPT
SBP(1,2,3)	112.58 (10.96)	116.35 (14.12)	112.08 (11.61)	114.17 (14.02)	139.20^a, ***^ (16.00)	112.17 (11.07)
DBP(1,2,3)	71.08 (5.50)	73.50 (8.65)	71.15 (7.28)	72.20 (9.03)	92.13^a, ***^ (11.14)	71.77 (7.40)
HR(1,2,3)	67.46 (11.43)	66.42 (10.10)	66.19 (10.23)	74.03 (11.73)	73.80 (13.71)	70.07 (10.53)
DASS(1,2,3)	15.58 (4.74)	15.54 (4.69)	11.77^b, ***^ (12.27)	15.07 (4.19)	16.50 (4.86)	12.27^b, ***^ (5.24)

SBP, systolic pressure; DBP, diastolic pressure; HR, heart rate; DASS-21, stress subscale. Standard deviations are in parentheses. ^a, ***^ Stress > Control at the corresponding measurement point, *p* < 0.001. ^b, ***^ DASS3 < DASS1 and DASS2, *p* < 0.001.

There were significant effects for systolic blood pressure. The 2 (Group: Stress vs. Control) × 3 (Time: Pre-SECPT, During SECPT, and Post-SECPT) mixed ANOVA showed a main effect of Group, *F*(1, 54) = 7.40, *p* = 0.01, ${\;\eta }_{p}^{2}$ = 0.12, a main effect of Time, *F*(2, 108) = 66.29, *p* < 0.001, ${\;\eta }_{p}^{2}$ = 0.55, and a Group × Time interaction, *F*(2, 108) = 31.91, *p* < 0.001, ${\;\eta }_{p}^{2}$ = 0.37.

There were also significant effects for diastolic blood pressure. The mixed ANOVA showed a main effect of Group, *F*(1, 54) = 14.62, *p* < 0.001, ${\;\eta }_{p}^{2}$ = 0.21, a main effect of Time, *F*(2, 108) = 65.12, *p* < 0.001, ${\;\eta }_{p}^{2}$ = 0.55, and a Group × Time interaction, *F*(2, 108) = 36.10, *p* < 0.001, ${\;\eta }_{p}^{2}$ = 0.40.

Both results (systolic and diastolic blood pressure) indicate that the manipulation significantly activated the sympathetic nervous system, with higher blood pressure during hand submersion in ice water in the Stress group relative to the Control group.

For heart rate, there was only a main effect of Group, *F*(1, 54) = 4.77, *p* = 0.033, ${\;\eta }_{p}^{2}$ = 0.08. There was neither a main effect of Time, *F*(2, 108) = 2.80, *p* = 0.065, ${\;\eta }_{p}^{2}$ = 0.05, nor a Group × Time interaction, *F*(2, 108) = 1.16, *p* = 0.318, ${\;\eta }_{p}^{2}$ = 0.02.

For the stress subscale of the DASS-21, there was a main effect of Time, *F*(2, 108) = 36.59, *p* < 0.001, ${\;\eta }_{p}^{2}$ = 0.40, but neither a main effect of Group, *F*(1, 54) = 0.09, *p* = 0.762, ${\;\eta }_{p}^{2}$ = 0.002, nor a Group × Time interaction, *F*(2, 108) = 1.13, *p* = 0.327, ${\;\eta }_{p}^{2}$ = 0.02. Participants reported lower stress at the final measurement (DASS3) than at the previous two measurements. This reduction was observed in both groups.

#### Cortisol levels

In the Test Phase, participants immersed one hand in cold water for about 2 min. The difference in temperature between the control and stress conditions was significant (*M*_cold_ = 2.43 °C, *SD* = 0.83 vs. *M*_warm_ = 30.43 °C, *SD* = 1.49, *t*(54) = 88.13, *p* < 0.001, *d* = 23.62). The low temperature induced physiological responses of the Sympathetic Nervous System, and along with the psychosocial elements of the task (e.g., doing mental arithmetic with an evaluator observing), induced the release of cortisol into the bloodstream.

**Figure 3:**
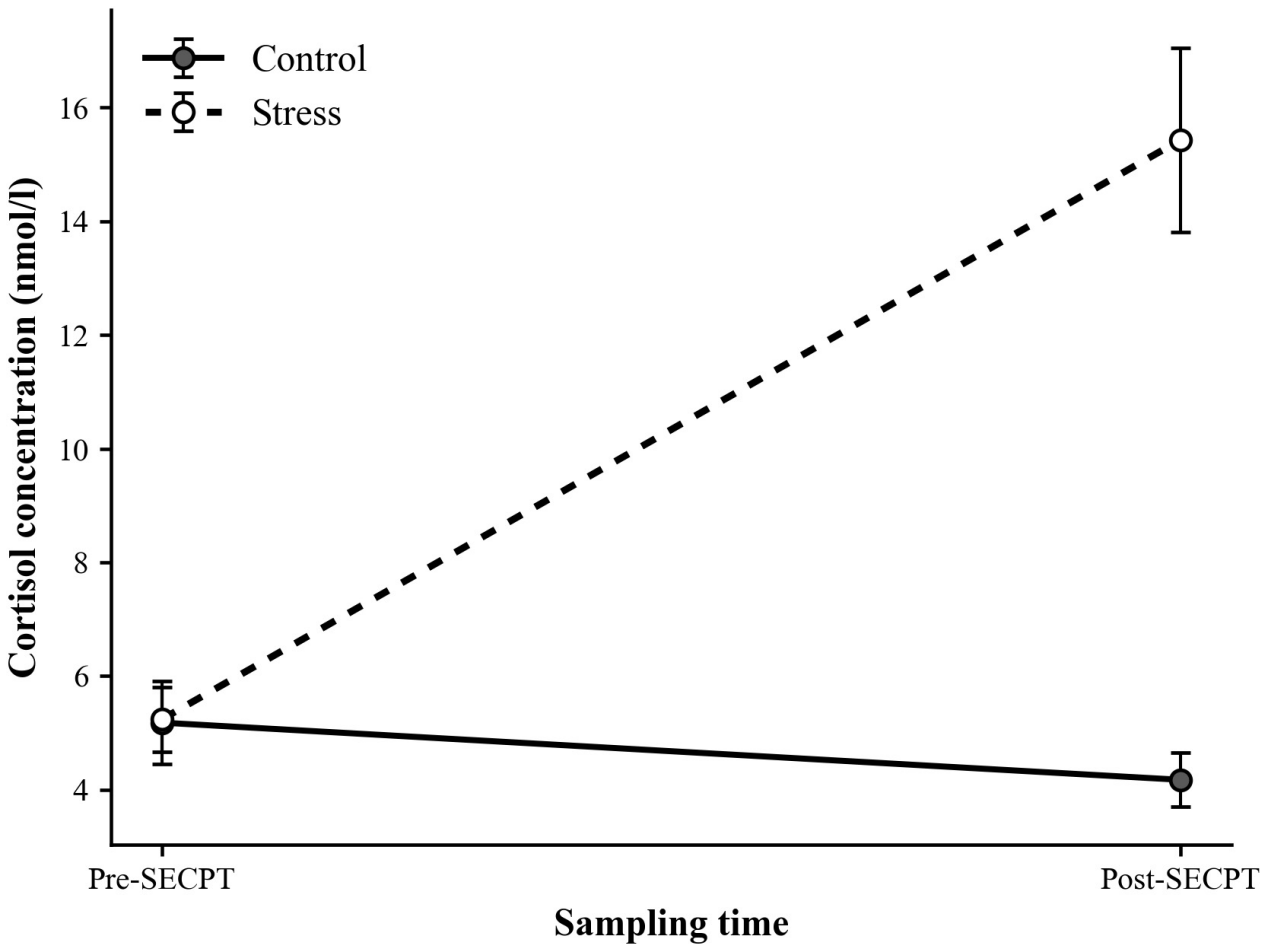
Salivary cortisol levels. The cortisol concentration increased significantly in participants in the stress condition, but it did not change in those in the control condition. Bars represent the standard error of the mean.

A 2 (Condition: control vs. stress) × 2 (Time: pre-SECPT vs. post-SECPT) mixed ANOVA on salivary cortisol concentration showed a strong interaction, *F*(1, 54) = 40.52, *p* < 0.001, ${\;\eta }_{p}^{2}$ = 0.43. This interaction indicated that cortisol concentration increased markedly from pre- to post-test in the stress condition (*p* < 0.001), whereas the control condition showed a small decrease across time (*p* = 0.032). In addition, cortisol concentrations did not differ between groups at baseline (*p* = 0.950), but were significantly higher in the stress condition than in the control condition at post-test (*p* < 0.001). Figure 3 illustrates this result, which indicates that the SECPT protocol produced the expected effect in terms of inducing an increase in salivary cortisol.

#### Performance on the final cued-recall test

**Figure 4:**
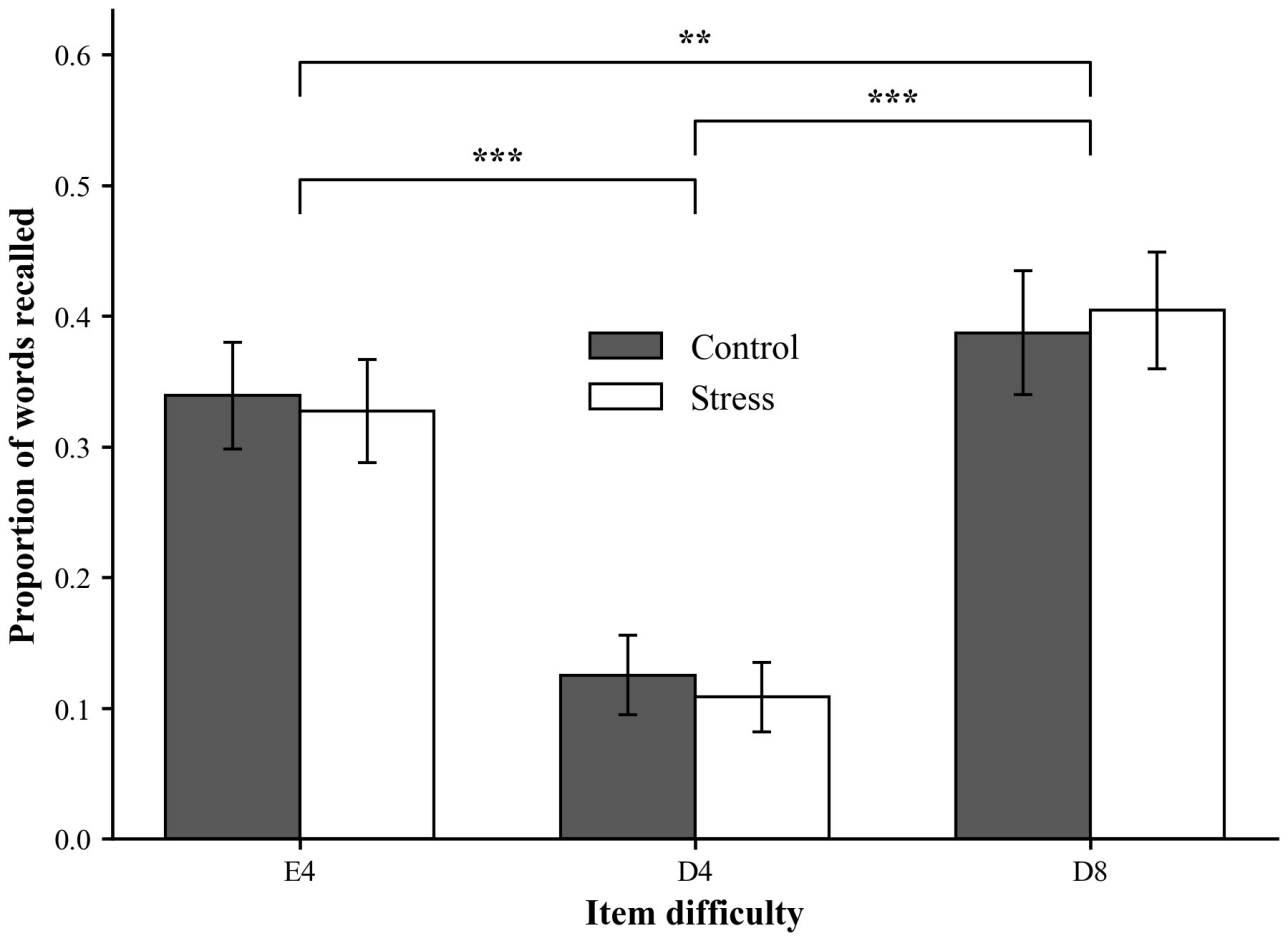
Proportion of word pairs recalled as a function of item difficulty and the group to which the participant was allocated. There was only an effect of difficulty, with fewer pairs recalled in the D4 condition than in the E4 and D8 conditions. Bars represent the standard error of the mean. ** *p* < 0.01; *** *p* < 0.001.

The two main results refer to the participants' performance on the final cued-recall test. Out of the 48 Swahili-Portuguese word pairs, how many Portuguese words did the participant recall when presented with the Swahili cue 48 h after learning and under stress? Figure 4 illustrates the pattern of results in relation to item difficulty (E4, D4, D8) and Group (control vs. stress).

A mixed factorial ANOVA 3 (Difficulty: E4, D4, D8) × 2 (Group: control, stress) on the proportion of words recalled showed only a main effect of the Difficulty factor, *F*(2, 108) = 91.22, *p* < 0.001, ${\;\eta }_{p}^{2}$ = 0.63. There was neither a main effect of Group nor a Difficulty × Group interaction, *Fs* < 1, *ps* > 0.70. As also observed in the Encoding Phase, memory performance in the Test Phase was low, with a recall rate below 50% (24 word pairs) in all Groups.

#### Performance on the final cued-recall test (conditionalized)

The observed pattern of a significant interaction between item difficulty and stress was more strongly observed when the analysis was conditionalized (Klier and Buratto, 2023). In this type of analysis, the denominator in the calculation of the number of recalled items is restricted only to items recalled at least once in the initial learning cycles. By analyzing the data in this way, we emphasize the items that were, in fact, learned 48 h earlier, unlike the non-conditionalized analysis, where the number of items recalled in the final test is counted relative to the total expected items (48 pairs). The conditionalized analysis allows focusing on stronger memory traces, which were indeed encoded in the initial stage. Such traces may be more resistant to the effects of stress, so a Group effect was not expected in this analysis. However, an interaction similar to that observed in Klier and Buratto (2023) is expected. More importantly, it was expected that the pattern in the D8 condition would be similar to that in the E4 condition, as the D8 items became functionally “easy” (Figure 2) and, therefore, should behave similarly to the easy items (E4).

**Figure 5:**
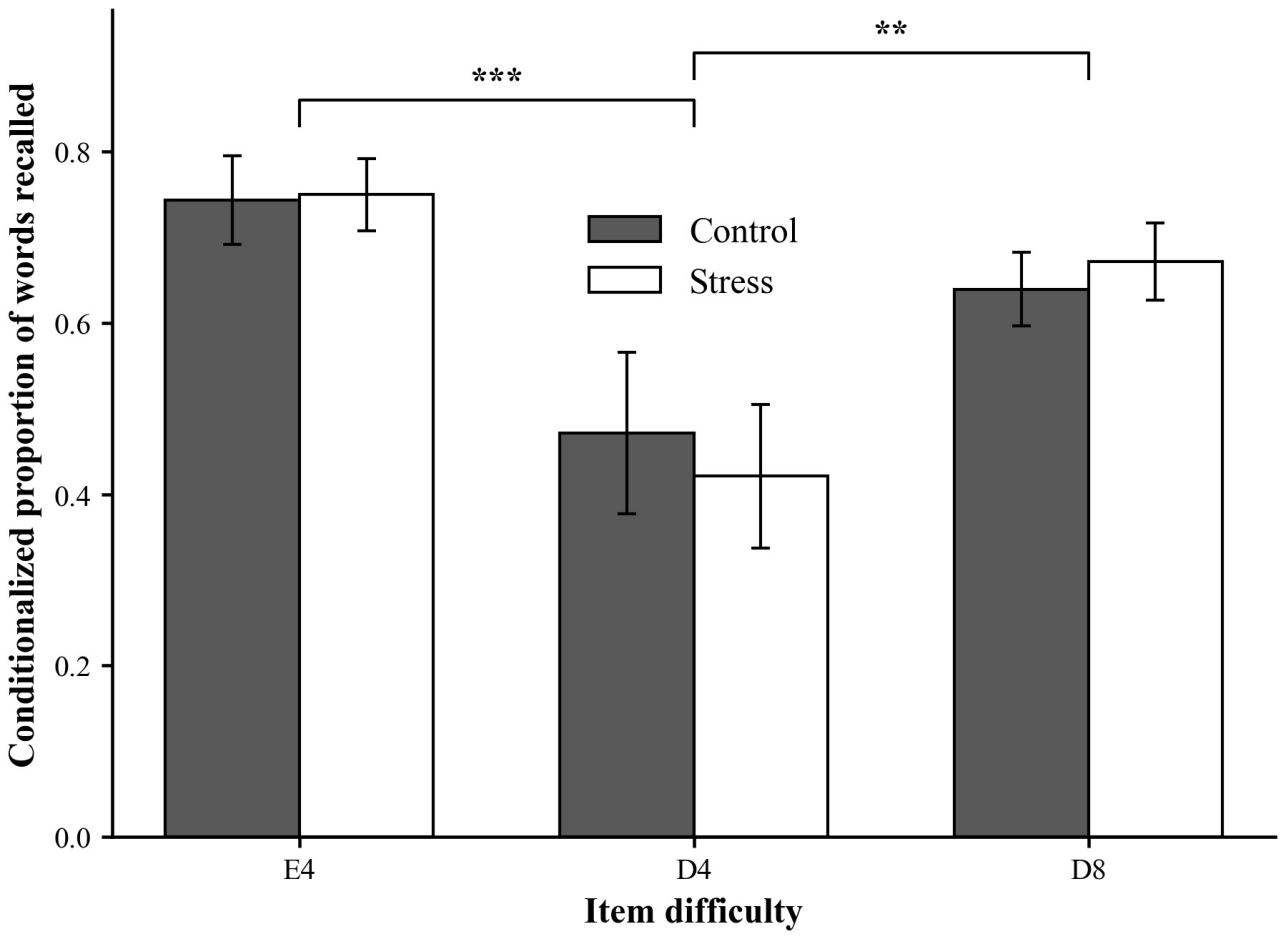
Conditionalized proportion of word pairs recalled as a function of item difficulty and the group to which the participant was allocated. This analysis was conditionalized on items that were recalled at least once during the Encoding Phase. There was an effect of difficulty, with less recall for D4 items than E4 and D8 items. The patterns for E4 and D8 in relation to the stress manipulation were similar. Bars represent the standard error of the mean. ** *p* < 0.01; *** *p* < 0.001.

A 3 (Difficulty: E4, D4, D8) × 2 (Group: control, stress) mixed factorial ANOVA on the conditionalized proportion of words recalled revealed a main effect of the Difficulty factor, *F*(2, 108) = 13.89, *p* < 0.001, ${\;\eta }_{p}^{2}$ = 0.20. There was no main effect of Group, nor a Difficulty × Group interaction, *Fs* < 1, *ps* > 0.77. Figure 5 illustrates these results.

#### Cortisol responders vs. non-responders

We also classified participants into cortisol responders (responders) and non-responders. The idea is that individuals who released more cortisol as a result of the stress induction protocol might show greater impairment in the final memory task. To be classified as a responder, the participant must produce at least 2.5 nmol/L more cortisol after the stress induction protocol relative to the baseline condition.

A contingency table showed that 23 of the 30 participants in the Stress group were cortisol responders. In contrast, only 1 of the 26 participants in the Control group were responders, χ^2^(1) = 27.26, *p* < 0.001. Upon reanalyzing the cued recall and conditional cued recall data, there was no change in the results.

Reanalyzing the final recall data with responder status as the between-subjects factor did not change the overall pattern of results. The 3 (Difficulty) x 2 (Responders) ANOVA on the proportion of words recalled showed only a main effect of Difficulty, *F*(2, 108) = 90.67, *p* < 0.001, ${\;\eta }_{p}^{2}$ = 0.63. There was neither a main effect of the Responders factor nor a Difficulty × Responders interaction, *F*s < 1, *p*s > 0.66.

In the conditional analysis, the result was similar. The 3 (Difficulty) × 2 (Responders) ANOVA on the proportion of words recalled showed only a main effect of Difficulty, *F*(2, 108) = 14.22, *p* < 0.001, ${\;\eta }_{p}^{2}$ = 0.21. There was neither a main effect of the Responders factor nor a Difficulty × Responders interaction, *F*s < 2, *p*s > 0.22.

Therefore, differences in cortisol release as a consequence of the stress induction protocol do not explain the results observed in the final memory test.

## Discussion

In this study, participants were instructed to learn 48 Swahili-Portuguese word pairs for a memory test 48 h later. On the day of the memory test and before retrieval, half of the participants underwent a stress induction protocol, and the other half did not. The protocol was successful in that it increased the concentration of cortisol only in participants in the Stress Group and not in participants in the Control Group. Furthermore, physiological measures, such as heart rate and systolic pressure, also increased only when participants submerged their hand in cold water, which was part of the stress protocol, and not when they submerged it in warm water, which was part of the control protocol.

In addition, participants were acclimated to the study environment (10 min) before the stress induction protocol and waited 25 min between the onset of the stress protocol and the memory test (so that circulating cortisol reached its expected peak). Then they performed only one evaluated cognitive task (cued recall). Participation in the experiment was in the afternoons, a period when cortisol levels are more stable. All these characteristics of the experimental procedure are important for stress induction and are recommended by experts (Shields, 2020).

If stress induction was successful, why no impairments in participants' recall were observed? In this study, we chose to use a strong encoding strategy, Retrieval Practice, in all conditions. When this strategy was used, the deleterious effects of stress on memory were reduced or disappeared (Mihaylova et al., 2025; Smith et al., 2016, 2018b). Therefore, there was no expectation of strong stress effects on final recall (traditional or conditionalized). What was expected was a modulation of stress according to item difficulty, as in Klier and Buratto (2023).

In that study, it was observed that stress decreased recall for easy items and increased it for difficult items. We did not observe these results in the present study. We can rule out some possible confounding factors that could affect the result. For example, in the Encoding Phase, participants in the Stress Group showed a small, but consistent, advantage in learning compared to participants in the Control Group. These initial differences could explain the results in the final test where recall in E4 and D8 was slightly higher in the stress condition than in the control condition. To resolve this issue, we added the final learning cycles of E4 and D8 as covariates in the 3 x 2 ANOVAs conducted. The results were not altered. Therefore, it is unlikely that the small differences in encoding were responsible for the small differences observed in the final test.

Another relevant difference between the present study and Klier and Buratto (2023) concerns the retention interval. In the previous study, the final test occurred seven days after encoding, whereas in the present study it occurred after 48 h. This difference may be critical because the impact of stress on retrieval likely depends on the accessibility of the memory trace at the time of testing. Longer retention intervals are expected to reduce retrieval strength, and under these conditions, acute stress may be more likely to impair retrieval. In contrast, after a shorter interval, items strengthened through repeated retrieval practice—especially in the E4 and D8 conditions—may retain sufficient retrieval strength to resist the disruptive effects of stress. Thus, the absence of a Stress × Difficulty interaction in the present study may reflect, at least in part, the shorter retention interval used here.

We acknowledge that further research may be necessary to determine whether this absence of interaction is a robust phenomenon or a byproduct of the current experimental constraints.

### Self-report questionnaire

Another factor that could affect the results was the DASS-21 self-report questionnaire (stress subscale). In the original study, Klier and Buratto (2023) used the BAI (Beck Anxiety Inventory) to measure participants' anxiety levels throughout the study. In Klier and Buratto (2023), the BAI showed consistent results, with higher anxiety in the stress condition than in the control condition. Here, the DASS-21 questionnaire showed a result that indicates higher anticipatory stress and stress during the protocol compared to the end of the experiment. However, such behavior was similar for both the Control Group and the Stress Group. This may indicate that participants in the Control Group felt (i.e., subjective judgment) as threatened or stressed as participants in the Stress Group. If this is true, then this would weaken the stress manipulation (strongly validated by cortisol, but weakly validated by subjective self-report measures). Alternatively, Control Group participants who scored high on the DASS-21 may have behaved similarly to individuals subjected to the stress protocol. These individuals would be subjectively stressed and, therefore, might behave as such in terms of memory performance. These speculations need to be better tested. Shields (2020) recommends the use of some questionnaires that can be used in future studies (e.g., Primary Appraisal Secondary Appraisal scale, Stanford Acute Stress Reaction Questionnaire). However, these instruments are awaiting validation in Brazil.

### Repetition of difficult items

The manipulation of repeating difficult items (D8) was successful in that it functionally created a set of items with memorability values close to those of easy items (E4). In this way, we avoided the confounding factor that difficult items (D4) would already have an initial disadvantage in the learning cycles, and this could influence the results in the final recall tests. The pattern of results in the recall tests was very similar between E4 and D8, both in the traditional analysis and in the conditionalized analyses. One interpretation is that, by being studied over eight cycles, the difficult items acquired properties of easy items (e.g., frequency) and, therefore, would behave functionally as easy items. In fact, in both conditions, there was a small advantage for the Stress Group over the Control Group.

### Participants' performance

A factor that could explain the failure to replicate this study, despite most manipulations producing the desired results, was the low performance of the participants both in the Encoding Phase and in the (non-conditionalized) Test Phase. On average, participants learned only 8 pairs of items in the final cycle out of a total of 48 pairs and recalled less than 50% of the pairs in the final test. This is a very low value, including easy pairs (i.e., with Swahili spelling and pronunciation similar to Portuguese). With such low levels of learning, it becomes difficult to conduct robust statistical tests in the Test Phase, when more items would have been forgotten 48 h after learning. This factor related to the participants may be linked to motivation (e.g., absence of any incentive for them to perform well) and the session duration (i.e., both sessions lasted about 60 min). Such motivational factors may have affected the participants' performance independently of their Group, reducing both the stress effect and the possibility of a Difficulty x Group interaction.

### Future directions

Future studies should further investigate the conditions under which stress interacts with retrieval practice and item difficulty to affect memory performance. One critical factor concerns the retention interval, as discussed above. Directly comparing short and long delays within the same experimental design would allow testing whether stress-related impairments emerge preferentially when retrieval strength has declined and performance becomes more dependent on momentary accessibility.

In addition, it will be important to examine how retrieval effort and encoding strength jointly constrain these effects. In the present paradigm, these factors were partially dissociated by manipulating item difficulty and practice dosage. Future studies could extend this approach by more systematically varying the number of successful retrievals across conditions, given the well-established role of retrieval success in strengthening memory traces.

Another relevant issue concerns the characteristics of the stress manipulation. Although the modified SECPT reliably increased cortisol, subjective stress did not clearly differentiate between groups. This raises the possibility that different components of the stress response may play distinct roles in modulating retrieval. Comparing protocols that vary in social-evaluative threat and unpredictability may help clarify this issue.

## Data Availability

The raw data supporting the conclusions of this article will be made available by the authors, without undue reservation.
